# Editorial: Current and future perspectives for controlled environment agriculture (CEA) in the 21st century

**DOI:** 10.3389/fpls.2023.1334641

**Published:** 2023-11-27

**Authors:** Kambiz Baghalian, Mohammad-Reza Hajirezaei, Tracy Lawson

**Affiliations:** ^1^School of Sustainable Environment, Writtle University College, Writtle, United Kingdom; ^2^Physiology and Cell Biology, Leibniz Institute of Plant Genetics and Crop Plant Research, Gatersleben, Germany; ^3^School of Life Science, University of Essex, Colchester, United Kingdom

**Keywords:** Controlled Environment Agriculture (CEA), vertical farming, food sustainability, food security, LED lighting applications

Controlled Environment Agriculture (CEA) is the production of crops under protected environments by optimizing climate and inputs (water, feed, chemicals, energy, labor, etc.). The aim is to maximize crop quality and quantity, extend the growing season and reduce inputs. However, with increasing costs of energy, labour, and consumables, major concerns in the 21st century exist around the sustainability of CEA. Fortunately, decades of innovation made by pioneers in areas such as breeding, greenhouse infrastructure, energy, computer science and more, have created the potential capacity to address these issues directly. In this Research Topic, we delve into the fascinating and dynamic world of CEA research and explore the latest research and innovative approaches that offer promise for improving sustainability features of CEA. Towards that aim, this Research Topic invited manuscripts focusing on multi-disciplinary approaches and novel technologies of CEA, summing up to five original research articles. The papers represent an eclectic mix that demonstrates the range of research aspects on CEA such as optimising environment (light quality, quantity, etc.), optimising crop growth strategies (indoor pest control) and the application of AI and computing technologies along with sensor development.

The highlights include Kim et al.‘s work who successfully developed a fuzzy logic-control system for small scale cultivation of sweet basil (*Ocimum basilicum* L.*).* Many previous studies have taken a rather similar approach but their proposed fuzzy systems are purely simulation with no actual implementation of the proposed fuzzy system. This makes it difficult to assess their actual efficiency of the proposed system. Another shortcoming from existing studies is that they propose no modification method to examine the vulnerable components of a given fuzzy logic design. Nevertheless, the new design by Kim et al. has successfully addressed this issue by implementing three inputs (temperature, humidity, and growth stage), along with seven outputs (fan, humidifier, heater 1, heater 2, red/blue/green LED light), and six fuzzy system elements. The developed fuzzy logic based automated system ensured that actuators operated properly according to sensors. Although this design supports small-scale operations, it potentially provides a route to designing similar systems, which can operate on a larger commercial scale.

The application of beneficial insects to provide biological control has become a routine practice in CEA. Li et al. explored beneficial insects and the application from a different and rather innovative angle, investigating the relative contribution of hoverflies (*Eupeodes corolla*) to pollination and aphid biological control in three crops grown under controlled environment, including melon (*Cucumis melo* L.), tomato (*Solanum lycopersicum* L.), and strawberry (*Fragaria* x *ananassa* Duch) (Li et al.). The overall outcome of this research showed how ugmentative hoverfly releases increase fruit set and crop yield while securing insecticide-free aphid pest control. To fully exploit the benefits of studies such as this for CEA, more research is required to investigate aspects such as release scheme, habitat management strategies and the integration with other natural enemies.

Other published papers in this Research Topic focused on different aspects of plant and light interactions (Ke et al.; Zhang et al.; Kang et al.). LEDs have become a crucial part of CEA industry and a fundamental element for vertical farming systems (VFS). The mainstream of crops grown in VFS are leafy vegetables and micro salads as they have a small root system and a high harvest index, allowing for crops to be grown to a small size whilst producing the highest yield by directing photo-assimilates to marketable organs (van Delden et al., 2021). However, Ke et al.‘s study has looked at the idea of extending the range of crops commercially grown if VFs, beyond the current mainstream of leafy vegetables and salads. This study explored the potential of dwarf tomatoes cultivation in VFs for commercial fruit production. Their study aimed to analyze the effects of photosynthetic photon flux density (PPFD) on fruit biomass radiation-use efficiency (FBRUE) in the dwarf tomato cultivar ‘Micro-Tom’ and to determine the suitable PPFD for enhancing the FBRUE under LED lighting at the reproductive growth stage. They also aimed to identify the effects of PPFD on the source strength and fruit sink strength of dwarf tomatoes during the reproductive growth stage. This study recommended 300 µmol m^−2^ s^−1^ PPFD for ‘Micro-Tom’ cultivation to improve the FBRUE at the reproductive growth stage. While this is one of the first studies elucidating the effects of PPFD on the source and fruit sink strengths of dwarf tomatoes in VFS, further research is required in future to assess the light quality and dynamic PPFD management for improving FBRUE and yield in ‘Micro-Tom’.

Another study conducted by Zhang et al., aimed to explore the potential of manipulating the light environment using LEDs. What makes this research more innovative is the fact that *Artemisia annova* is an outdoor crop and authors aimed to study the feasibility of its commercial production in CEA. Moreover, this study was looking to improve not only the plant biomass but also the production of Artemisinin, the active secondary metabolite, with commercial application in anti-malarial medicines. The results highlighted the potential of applying light treatments to increase trichome density in vegetative stage, which is one of the location for Artemisinin production. However, the trade-off between light effects on trichome initiation and overall plant growth need to be considered, as plant biomass decreased with increased trichome density. In a similar approach, Kang et al. described responses of sweet basil (*Ocimum basilicum* L.) grown under a base red/blue/green LED light with four supplemental UV-A intensity treatments (0, 10, 20, and 30 W·m^−2^) to see if UV-A radiation could improve both yield and quality (accumulation of beneficial phenolic compounds) of the crop. Overall, results indicated that the biomass production and accumulation of beneficial phenolic compounds can be improved under Mild UV-A radiation (10–20 W·m^−2^).

With the five papers in this Research Topic on **“Current and Future Perspectives for Controlled Environment Agriculture (CEA) in the 21st Century”**, we hope readers will gain further insights into the dynamic world of CEA, and the opportunities it can offer together with the challenges facing the sector. The advancement made in CEA has illuminated the path towards more sustainable food production and global food security. The papers presented in this Research Topic collectively emphasize the critical role of technology in supporting growth in CEA and highlights the significant role biological insights play in guiding technological development in the right direction. This certainly requires continued research and innovation to grow our understanding of how technology can support CEA. While implementing and optimizing new technological advancements holds great promise for the CEA sector, breeding crops, which are more suitable for such settings and understanding their interactions with environment is equally important and challenging. These efforts collectively can pave the way for a sustainable and food-secure future.

## Author contributions

KB: Writing – original draft, Writing – review & editing. M-RH: Writing – review & editing. TL: Writing – review & editing.
